# Limitations of molecular testing in combination with computerized tomographic for lung cancer screening

**DOI:** 10.1038/s41467-022-31419-9

**Published:** 2022-07-08

**Authors:** Frederic W. Grannis

**Affiliations:** Clinical Professor of Thoracic Surgery (retired), City of Hope National Medical Center, 1500 East Duarte Road, Duarte, CA 91010 USA

**Keywords:** Molecular medicine, Cancer screening

**arising from** Mathios et al. *Nature Communications* 10.1038/s41467-021-24994-w (2021)

Lung cancer is the most common cause of cancer death in both men and women; reduced mortality and improved long-term survival have been described in high-risk participants in computerized tomographic screening research programs. Mathios et al. present interesting data on a new ‘fragmentomic’ method, which they suggest might be valuable as a pre-test, to enhance the early detection of lung cancer^1^. The results from their study do not support the assertion that their molecular screening method is sensitive enough to detect slung cancer at a small size and early stage. If this or other molecular screening tests are used as ‘pre-testing’ to enrich the yield of CT screening, substantial numbers of individuals with early-stage lung cancer might be missed and fail to benefit from a more sensitive modality, the low-dose, non-contrast spiral CT scan.

Their data shows that the Delfi test, using a cut-point of 0.34 units, is elevated in most patients with clinical lung cancer.

The authors go on to propose that a commercial version of the Delfi test, might be useful in refining population lung cancer screening; serving as a pre-test, offered to the approximately fifteen million Americans who are at known high risk of lung cancer. Those with positive fragmentome levels would then move on to CT screening, while those with negative tests would not. The authors’ conclusions are puzzling, since they present no data on individuals who meet the eligibility criteria for lung cancer screening. The Centers for Medicare and Medicaid Services mandates that patients must be asymptomatic to be eligible for CT screening. 90% of the authors’ study subjects had symptoms suggestive for lung cancer and all had an abnormality on x-ray or CT scan.

The core goal of lung cancer screening is to detect disease at a small size and early stage, where cure is usually possible, but in the LUCAS group studied, most patients had advanced lung cancers (Stages 2–4); only 15 were in Stage 1, and only 8 had adenocarcinoma (with generally better prognosis). This stage mix is important; it is widely understood that, when patients present with symptoms of lung cancer, more than 80% have advanced-stage disease, and prospect of a cure is low. The fact that symptomatic LC is infrequently cured, provides the basis for population lung cancer screening of asymptomatic individuals.

The authors concede that Delfi scores were substantially lower in patients with small lung cancers, i.e., those less than 3 cm. in diameter, and those without lymph node metastases (pathologic Stage 1). Specifically, they report mean Delfi scores for stage 1 tumors at 0.34 units, precisely at the test’s cut-point, and state that stage I disease was more difficult to identify.

Also of concern, the authors provide very limited information on the diameter or volume of tumors in their study subjects. This is a very important limitation. CT screening for LC is effective in saving lives, because it can identify lung nodules as small as 2 mm. in diameter. In the 1999 ELCAP study more than 80% of lung CA were diagnosed and treated in Stage 1^2^. The 2006 IELCAP study, reported ten-year actuarial survival of 85% among patients with screen-detected lung cancer in Stage 1^3^. The median tumor diameters at baseline and annual repeat CT screening in I-ELCAP study subjects were 13 and 8.8 mm, respectively^4^. It is not clear that any of the cases documented in the Delfi study fell into this size category. The authors do not provide a table detailing the size of the T1 or stage 1 patients included in their experiment, except to observe that, eight of the nine tumors <2 cm in size (T1a) had DELFI scores higher than the median non-cancer population.

The data presented in this paper suggest that the pre-test proposed might be considerably less sensitive than CT screening itself. If substantiated in further research, this would imply that fragmentomic pre-testing not only would not add value; it could cause harm. Patients with small lung cancers who received false negative pre-test results would be ineligible or have delayed access to more sensitive CT scans, proven to save lives in multiple clinical trials.

Delfi is currently recruiting research participants into a new clinical study “DNA Evaluation of Fragments for Early Interception - Lung Cancer Training Study (DELFI-L101 Study) (DELFI-L101)”

This study, however, will again compare patients with a known diagnosis of lung cancer, with a case-control group of subjects who receive the Delfi test, but not a CT scan. The ability of molecular testing to refine lung cancer screening, by identifying small, pre-symptomatic, early-stage lung cancers, can only be determined in a prospective experiment that follows a cohort of high-risk individuals screened with both molecular testing and annual CT screening.
